# Global Spine Range of Motion in Patients With Adolescent Idiopathic Scoliosis Before and After Corrective Surgery

**DOI:** 10.7759/cureus.19362

**Published:** 2021-11-08

**Authors:** Yusuf Mehkri, Jairo Hernandez, Jessica L McQuerry, Johanna Carmona, Stephanie Ihnow

**Affiliations:** 1 Medicine, University of Florida College of Medicine, Gainesville, USA; 2 Surgery, University of Florida College of Medicine, Gainesville, USA; 3 Pediatric Orthopaedics, University of Florida College of Medicine, Gainesville, USA

**Keywords:** total spine range of motion, surgical treatment, spine mobility, motion analysis, adolescent idiopathic scoliosis (ais)

## Abstract

Given the importance of the spine in carrying out daily movements, adolescent idiopathic scoliosis (AIS) can significantly limit the range of motion (ROM). Severe forms of AIS are treated surgically, most commonly with posterior spinal fusion and instrumentation, which may also reduce spine ROM. This review is the first to describe the literature on total spine ROM in patients with AIS before and after corrective surgery. A systematic literature search was performed using PubMed and Google Scholar to identify articles reporting global spine ROM in AIS patients. Following the guidelines of the Preferred Reporting Items for Systematic Reviews and Meta-Analyses (PRISMA), 486 articles were initially identified. Two independent reviewers (YM and JH) assessed eligibility for inclusion.

A total of 11 articles fit the inclusion criteria. AIS in untreated patients seems to limit axial and coronal plane ROM based on the degree of curve severity, with more severe curves having less ROM. More research comparing total spine ROM in untreated AIS patients to that of healthy controls is needed. In those undergoing spinal fusions, the lowest instrumented vertebra and surgical approach appear to minimize further reductions in ROM; however, the findings are mixed. Vertebral body tethering (VBT) shows promising preliminary results in treating AIS while preserving motion; however, long-term outcomes have yet to be assessed for this novel procedure. The results of this systematic review suggest that further research is required before treatment strategies can be modified for surgically treating patients with AIS to take into account the effects of treatment on changes in spine mobility.

## Introduction and background

Scoliosis is a complex three-dimensional spinal deformity with both vertebral rotation and lateral curvature of at least 10° [1]. Idiopathic scoliosis comprises 80% of cases of the condition and a combination of genetic, hormonal, and biomechanical mechanisms contribute to onset [1-4]. Genome-wide association studies have identified many single-nucleotide polymorphisms associated with adolescent idiopathic scoliosis (AIS) [5-7], bringing us one step closer to elucidating the genetic basis behind this condition. AIS affects 1-4% of adolescents between the age of 10 and until skeletal maturity (defined as a Risser ‎≥4) [8]. The female to male distribution ratio increases with age from 1 to ‎≥8 [9].

The spine is the central axis for truncal rotation about the pelvis, which permits lateral bending and anteroposterior flexion-extension. Daily movements, sport, and physical activities involve complex functioning of the healthy spine in three dimensions. For example, forward flexion and axial rotation for picking up an object, and axial rotation, lateral bending, and forward flexion for throwing a ball fast. The pelvis and joints distal to the spine along the kinetic chain operate within expected excursions and application of joint forces to execute the whole movement. Spinal deformities like AIS restrict or change spine motion and pelvis orientation, thereby impacting distal joint excursions and load bearing to achieve the motion [10].

Given the importance of spine motion on the ability to execute everyday activities, there is a need to study how spinal deformities such as AIS affect spine motion, and more importantly, to see whether corrective surgery restores or further worsens spinal mobility. This is the first review article to describe the literature on total spine range of motion (ROM) in patients with AIS before and after corrective surgery.

## Review

Methods

**Figure 1:**
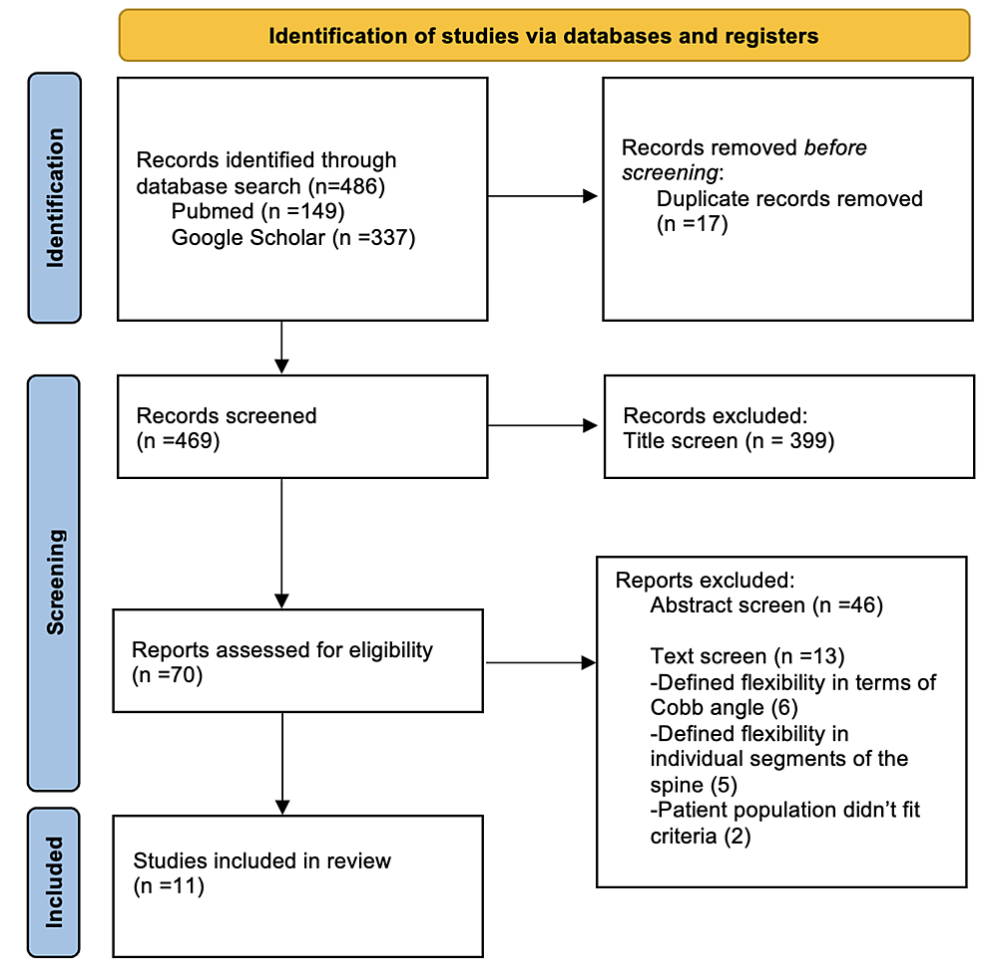
PRISMA flow diagram of the included studies. PRISMA, Preferred Reporting Items for Systematic Reviews and Meta-Analyses.

A systematic literature search was performed in August 2021 using PubMed and Google Scholar. Customized queries including the following keywords with AND/OR operators were entered into the search engines: AIS, adolescent idiopathic scoliosis, motion, and ROM. The search was limited to full-text original (retrospective or prospective) articles published in English after the year 2000. Papers that evaluated total spine ROM in patients with AIS before and/or after corrective surgery were included. Papers that focused on motion in specific spinal segments instead of total spine ROM were excluded (Figure 1).

Results

A total of 70 articles that fit inclusion criteria based on their title were retrieved. A total of 63 articles were excluded after screening abstracts and removing any duplicates. Most articles compared motion at specific spinal segments (e.g. proximal or distal to fusion) or focused on motion only in the cervical or lumbar spine. Reference lists were scanned for any additional articles that fit the criteria. A total of 11 articles published between 2002 and 2021 were ultimately included in this review.

One paper described total spine ROM before corrective surgery relative to the degree of spine curvature. The other 10 papers studied the change in spine motion in the front, lateral, and/or axial planes following fusion and instrumentation (Table 1). Although a quantitative meta-analysis could not be applied due to the heterogeneity of the studies included, the studies are qualitatively described to frame all that we know on the total spine ROM in the context of AIS.

**Table 1:** Summary of studies included in the review article. ROM, range of motion; LIV, lowest instrumented vertebra; ASF, anterior spinal fusion; PSF, posterior spinal fusion; VBT, vertebral body tethering.

Article	Objective	Reported preoperative or postoperative ROM	Patients: n (#F)
Eyvazov et al. (2017) [11]	How ROM changes with curve magnitude	Pre	58 (46F)
Engsberg et al. (2002) [12]	How ROM changes following fusion surgery	Post	30 (28F)
Lee et al. (2013) [13]	How LIV impacts ROM	Post	23 (18F)
Uehara et al. (2019) [14]	How LIV impacts ROM	Post	66 (61F)
Ohashi et al. (2020) [15]	How LIV impacts ROM	Post	151 (121F)
Udoekwere et al. (2014) [16]	How LIV impacts ROM	Post	47 (40F)
Danielsson et al. (2006) [17]	How ROM changes following fusion surgery (extended follow-up)	Post	156 (145F)
Engsberg et al. (2003) [18]	How ROM changes following different types of fusion surgery	Post	16 (13F) ASF/15 (13F) PSF
Helenius et al. (2002) [19]	How ROM changes following fusion surgery (extended follow-up)	Post	78 (67F)
Helenius et al. (2003) [20]	How ROM changes between different types of surgery (extended follow-up)	Post	78 (67F) Harrington/57 (48F) Cotrel
Pehlivanoglu et al. (2021) [21]	How ROM changes between different types of surgery	Post	21 (15F) VBT/22(16F) PSF

*Impact of AIS* *on Spine ROM*

Following a comprehensive literature search, only one study by Eyvazov et al. [11] investigated global spine ROM in adolescents with idiopathic scoliosis prior to corrective surgery. They recruited 58 patients with Lenke five curves and stratified them into two groups: curves <40 degrees and curves ≥40 degrees. Patients underwent ROM testing in the coronal, sagittal, and transverse planes, and the two groups were compared. The authors found curve severity to be associated with reduced axial and coronal ROM. In other words, axial rotation and lateral bending ROM decreased as curve severity increased. The lack of association with sagittal motion is interesting considering how dominant the lumbar spine is in the forward flexion and backward extension. The authors hypothesize that this may just have to do with the fact that the patients had different baseline activity levels and that they were also evaluated at different time points throughout the day, both of which can affect flexibility in the sagittal plane. Further research is required to see if these results can be replicated with a greater sample size while controlling for factors such as gender, activity level, and time of testing.

AIS has also been found to impact spine ROM by creating significant asymmetry in the coronal plane. This finding was described by two separate studies [12,18] where they noticed that AIS patients with right thoracic curves had significantly greater left lateral flexion. This would lead us to believe that patients with levoscoliosis would have significantly greater right lateral flexion; however, this has not been reported yet in the AIS literature.

There is a clear need for more research on how untreated AIS affects global spine ROM specifically when compared to normal controls. AIS-related changes in spinal mobility may be associated with early-onset back pain and disc degeneration [22,23] and should be taken into account when creating a treatment plan. Currently, emphasis is placed on curve correction for prevention of deformity progression, restoration of sagittal and coronal balance, and avoidance of cardiopulmonary issues in the future while maintaining as many motion segments as possible. Through a better understanding of how AIS can affect spinal mobility, we can guide treatment decisions and surgical planning to improve the quality of life in these patients.

Spine ROM after corrective surgery

Impact of Spinal Curvature

Only one study has analyzed how the curvature of a postoperative scoliotic spine can affect mobility. Helenius et al. [19] followed 78 patients for 21 years and conducted triplanar spine ROM testing. Although they did find reductions in motion in all planes (most significantly in lateral bending), they found no correlation between curve severity following fusion surgery and spine mobility. More research is required to specifically study how the degree of curve correction correlates with changes in mobility.

Impact of Lowest Instrumented Vertebra

Five studies investigated the impact of the lowest instrumented vertebra (LIV) on spine ROM. Engsberg et al. [12] evaluated triplanar ROM in 30 patients who underwent posterior, anterior, or combined anteroposterior spinal fusion surgery at one and two-year follow-ups. They found significant decreases in both left and right lateral flexion with left-right asymmetry (left lateral flexion being significantly greater) that was maintained from the preoperative visit. Of note, they were not able to replicate the postoperative left-right asymmetry in a future study [18]. They also found significant reductions in the forward flexion and trunk rotation following fusion surgery that was maintained at the two-year follow-up. Interestingly, no correlation was found between the number of levels fused, LIV/highest instrumented vertebra (HIV), and any of the aforementioned reductions in ROM.

Lee et al. [13] specifically investigated the impact of LIV on spine motion one year after surgery. They took 23 patients who underwent posterior spinal fusions and stratified them into two groups: those who were fused to the L1-L2 level and those fused further down to L3. They found that although both groups saw significant reductions in transverse rotation following surgery, only the L3 group saw losses in coronal motion. Specifically, those who were fused to the L3 level saw reductions in lateral bending following surgery that were significantly greater than those who were only fused to the L1-L2 level.

Uehara et al. [14] conducted a similar study comparing LIV on spine motion. They focused specifically on changes in sagittal motion using the fingertip-to-floor distance (FFD) test preoperatively and at two-year follow-up. With a sample size of 66 patients, they showed that FFD increased significantly as the LIV moved inferiorly from T11-T12 down to L3.

Udoekwere et al. [16] confirmed these results by evaluating triplanar ROM at one and two-year follow-ups in patients who underwent posterior spinal fusions. They classified 47 patients into five groups based on LIV (T12, L1, L2, L3, and L4). Although they saw significant reductions in all three planes postoperatively, only reductions in the forward flexion were correlated with more distal LIVs.

Ohashi et al. [15] built upon the findings of the previous three studies with a larger sample size (151) and longer follow-up time (10 years). They stratified the patients into either a thoracic fusion group (LIV at L1 or above) or a thoracic/lumbar fusion group (LIV at L2 or below). Evaluating both coronal and sagittal motion, they found significant reductions in both planes for both groups after 10 years. More importantly, the thoracic/lumbar fusion group, which had a more distal LIV, showed significantly greater reductions and recorded more patients with substantial reductions in motion.

Impact of Length of Fusion

One study directly investigated the effect of length of fusion on spine motion. Danielsson et al. [17] conducted a large-scale study with 156 surgically treated patients who were evaluated for their triplanar ROM 20 years postoperatively and compared to matched controls. As expected, ROM in all planes was significantly reduced compared to controls. When it came to the length of fusion, specifically, only lumbar mobility was negatively affected.

Three studies are the first to investigate the effects of the surgical approaches on functional outcomes. Helenius et al. [20] compared the effects of two types of posterior instrumentation on spine mobility. One group of 78 patients treated with Harrington instrumentation and another group of 57 treated with Cotrel-Dubousset instrumentation were followed for 21 and 13 years, respectively. They found a significantly higher number of patients with abnormal backward extension and lateral bending in the Harrington group. The authors did fail to address whether the large difference in follow-up time may have influenced these findings.

Similarly, Engsberg et al. [18] carried out a prospective evaluation of ROM in 31 AIS patients undergoing either a posterior or anterior surgical fusion. As the authors expected, the posterior group showed a greater loss in spine motion at two-year follow-up. Although both groups saw reductions in motion following surgery, the anterior group had significantly greater coronal, sagittal, and transverse (left rotation only) motion. Before concluding that anterior surgical fusions are superior from a functional standpoint, it is important to note that these results are confounded by the fact that the anterior group had shorter fusions and higher LIVs. The authors tried to control for this in a subanalysis using two major assumptions (detailed in their manuscript) and a smaller sample size; however, further research is still needed to adequately answer this question.

Recently, Pehlivanoglu et al. [21] conducted the first study comparing the functional outcomes of anterior vertebral body tethering (VBT) and posterior spinal fusion (PSF). The VBT group was age-gender-instrumented level and minimum follow-up duration matched with the PSF group, with 21 and 22 patients, respectively. The authors reported that VBT yielded significantly superior flexibility in terms of anterior and lateral bending compared to the PSF group. ROM in the sagittal, coronal, and transverse planes was also significantly increased in the VBT group. Although the results make VBT an incredible option for preserving motion, a major limitation of the study is its retrospective nature and the differences in PSF versus VBT indications for AIS patients in regards to preoperative curve flexibility and magnitude.

## Conclusions

This review of the current literature on spine ROM in patients with AIS before and after corrective surgery brings to light the many gaps that still need to be filled. We know that curve severity prior to treatment may predict losses in axial and coronal plane ROM and that patients may have left-right lateral bending asymmetry based on the direction of their curve. However, we have yet to take these changes in mobility into account when surgically treating these patients and have thus far placed the greatest emphasis on maximizing curve correction - a metric that has yet to be proven to improve functional outcomes.

The literature on postoperative changes in global spine mobility is growing. One study found no correlation between Cobb angle and mobility in any plane and there are mixed findings on the impact of a more distal LIV. Unfortunately, only four studies have compared different surgical approaches based on functional outcomes for patients with AIS. Given the importance of restoring mobility and advancements in the surgical treatment of severe AIS, there is an even greater need to study how new technology and instrumentation may affect mobility.

A better understanding of spine mobility in untreated AIS and after operative correction with modern surgical options would help guide treatment decisions, optimal level selection, and pre-surgical discussion to improve the quality of life of patients with AIS.
